# An Experimental and Theoretical Investigation of the Electronic Structures and Photoelectrical Properties of Ethyl Red and Carminic Acid for DSSC Application

**DOI:** 10.3390/ma9100813

**Published:** 2016-10-01

**Authors:** Chaofan Sun, Yuanzuo Li, Peng Song, Fengcai Ma

**Affiliations:** 1 College of Science, Northeast Forestry University, Harbin 150040, Heilongjiang, China; cfsunnefu@126.com; 2 Department of Physics, Liaoning University, Shenyang 110036, Liaoning, China; pengsong@lnu.edu.cn (P.S.); fengcaima@lnu.edu.cn (F.M.)

**Keywords:** photoelectric conversion efficiency, density functional theory, excited state, chemical reactivity parameters, dye-sensitized solar cell

## Abstract

The photoelectrical properties of two dyes—ethyl red and carminic acid—as sensitizers of dye-sensitized solar cells were investigated in experiments herein described. In order to reveal the reason for the difference between the photoelectrical properties of the two dyes, the ground state and excited state properties of the dyes before and after adsorbed on TiO_2_ were calculated via density functional theory (DFT) and time-dependent DFT (TDDFT). The key parameters including the light harvesting efficiency (LHE), the driving force of electron injection ($\Delta G^{\mathrm{inject}}$) and dye regeneration ($\Delta G^{\mathrm{regen}}$), the total dipole moment ($\mu_{\mathrm{normal}}$), the conduction band of edge of the semiconductor ($\Delta E_{\mathrm{CB}}$), and the excited state lifetime (τ) were investigated, which are closely related to the short-circuit current density ($J_{\mathrm{sc}}$) and open circuit voltage ($V_{\mathrm{oc}}$). It was found that the experimental carminic acid has a larger $J_{\mathrm{sc}}$ and $V_{\mathrm{oc}}$, which are interpreted by a larger amount of dye adsorbed on a TiO_2_ photoanode and a larger $\Delta G^{\mathrm{regen}}$, excited state lifetime (τ), $\mu_{\mathrm{normal}}$, and $\Delta E_{\mathrm{CB}}$. At the same time, chemical reactivity parameters illustrate that the lower chemical hardness (*h*) and higher electron accepting power (ω^+^) of carminic acid have an influence on the short-circuit current density. Therefore, carminic acid shows excellent photoelectric conversion efficiency in comparison with ethyl red.

## 1. Introduction

Solar energy, as a clean and renewable energy, has many advantages including inexhaustibility, no pollution, and large-scale applications. In recent decades, how to effectively use solar energy has become the focus of researchers at domestic and international levels. Since 1991, Grätzel et al. [1] introduced the nanocrystalline porous electrode with a great ratio surface area and an organic electrolyte to a dye-sensitized solar cell (DSSC) for the first time. Its photoelectric conversion efficiency (PCE) reached 7.9%, and this technology has opened new doors to effectively utilizing solar energy.

Generally, the DSSC consists of four parts [2,3]: a nanocrystalline photoanode, a redox electrolyte, a counter electrode, and dye, of which the dye is crucial to determine the PCE of the DSSC. The dyes can be divided into metal-bearing, metal-free, and natural dyes. Up to now, the PCE of DSSCs based on ruthenium polypyridine has exceeded 10% [4], and that of DSSCs with zinc porphyrin complexes have surpassed 12% under standard global AM 1.5 solar conditions [5,6] However, the ruthenium dyes have such disadvantages: it is rare and expensive, it has relatively low extinction coefficients, it only absorbs visible light, and dye aggregates on the semiconductor. This has limited the application of metal-bearing dyes for DSSCs [7]. Recently, much research has focused on the study of metal-free organic DSSCs due to the rich raw materials, flexible molecular design, easy synthesis, low cost [8,9,10,11,12,13,14], and organic optoelectronic materials [15,16]. It is worth noting that Yao and co-workers [11] synthesized a metal-free organic dye (C281), which showed over 80% external quantum efficiency in a broad spectral range from 480 to 735 nm, and a high PCE of 13.0% under irradiance of simulated AM 1.5G sunlight (100 mW·cm^−2^). Moreover, Gao et al. [12] designed and synthesized three novel oligothiophene-linked phenothiazine dyes JY31, JY32, and JY33 by introducing alkyl chains on oligothiophene π-bridge and 4-butoxyphenyl group as the secondary donor, which significantly improved the open-circuit voltage and short-circuit current density, and the highest PCE for JY33 was 7.48%. Besides, Karlsson and coworkers [13] synthesized and tested a series of metal-free organic dyes with a core phenoxazine chromophore as sensitizers in DSSCs, and the results indicated that a dye with a furan-conjugated linker showed a shorter lifetime relative to dyes with the acceptor group directly attached to the phenoxazine. In addition, natural dyes such as chlorophylls [17,18,19], flavonoids [20,21], anthocyanins [22,23], and carotenoids [24,25] have also been applied to the research and development of DSSC owing to their environmental friendliness, relative abundance, easy preparation, and large absorption coefficients’ invisible region [26,27]. Kumara et al. [28] reported the research of black tea waste extract (BTE) as a potential sensitizer, and the DSSC sensitized with pigment complexes of BTE showed a PCE of 0.20%, while a significant increase (η = 0.46%) was observed when the pH of the pigment solution was lowered. Li and co-workers [29] investigated three natural dyes (*Forsythia suspensa*, Herba Violae, and corn leaf) as potential sensitizers, and the highest PCE was 0.96%, with open circuit voltage of 0.66 V, a short-circuit current density of 1.97 mAcm^−2^, and a fill factor of 0.74 among the three DSSCs.

In recent years, quantum chemical methods have become a feasible means to reveal the relationship between structures and properties of dye molecules, which provide a reliable theoretical basis for the rapid screening of highly efficient dye molecules [30,31]. Many researchers have succeeded in predicting the photoelectric properties of dyes and organic molecules based on the quantum chemical methods [32,33,34,35,36,37]. Zhang and collaborators presented a systematical investigation on the key parameters including the open circuit voltage and short-circuit current density of two dyes (1 and 2) based on density functional theory (DFT) and time-dependent DFT (TD-DFT) calculations, and the results showed that the insertion of phenyl ring in 2 enlarged the distance between the dye cation hole and the semiconductor surface, and made the benzothiadiazole (BTDA) unit far away from the semiconductor, resulting in a decreased charge recombination rate compared with that of 1. Feng and co-workers [38] investigated the aggregation effects of two organic dyes (WS-2 and WS-6) via DFT, TD-DFT, and density functional tight binding (DFTB) methods, implying that the aggregation had an greater influence on emission spectra compared with absorption spectra, and stronger aggregation induced larger intermolecular electronic coupling. In addition, Zarate et al. [39] reported a computational investigation about the role of the donor motif in the photo-injection mechanism displayed from a series of A-bridge-D structured dyes adsorbed on a (TiO_2_)_15_ anatase cluster in the DFT framework with the B3LYP, PW91, PBE, M06L, and Cam-B3LYP functionals, which successfully predicted the efficiency of the studied dyes in DSSC devices.

Ethyl red, as a kind of non-cyanine dye, possesses a simple structure and has a low synthetic cost, and it has not been used in the field of DSSCs according to our current knowledge. Moreover, carminic acid, often used as colorant in food and cosmetics or pigment for painters, has a wide range of raw material sources and does no damage to the environment. In this work, we selected these two dyes as sensitizers to investigate the optical and electrical properties of DSSCs in experiments aiming to explore the relationship between molecular structures and photoelectric properties. In order to analyze the experimental results in depth, the absorption spectra, electronic properties, and energy gaps of the two dyes before and after absorption on TiO_2_ were calculated via DFT and TD-DFT. The key parameters of the dyes absorbed on TiO_2_ were investigated to reveal the intrinsic reason for the difference in the PCE of the two dyes, and those parameters are closely related to the short-circuit current density ($J_{\mathrm{sc}}$) and open circuit voltage ($V_{\mathrm{oc}}$), including the light harvesting efficiency (LHE), the driving force of electron injection ($\Delta G^{\mathrm{inject}}$) and dye regeneration ($\Delta G^{\mathrm{regen}}$), the total dipole moment ($\mu_{\mathrm{normal}}$), and the conduction band of edge of the semiconductor ($\Delta E_{\mathrm{CB}}$). In addition, the excited state lifetime (τ) and total static first hyperpolarizability of the two dyes were calculated. The three-dimensional (3D) real-space analysis method was adopted to describe the charge transfer process in the dye/TiO_2_ complexes. Finally, the chemical reactivity parameters of the two dyes including electron affinity (*A*), ionization potential (*I*), chemical hardness (*h*), electrophilicity index (ω), electron donating power (ω^−^), and electron accepting power (ω^+^) were calculated. The elaborated calculations will provide a basis for explaining the experimentally different photoelectrical properties between the two dyes and develop the potential utility in DSSCs.

## 2. Experimental and Theoretical Methods

### 2.1. Experiment

**Figure 1:**
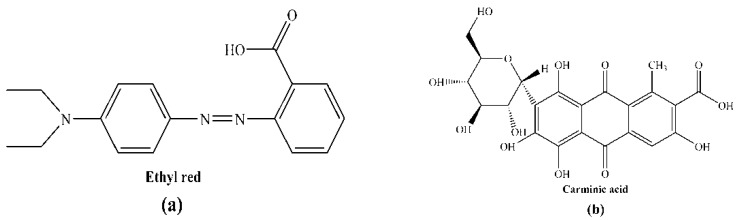
Chemical structures of (**a**) ethyl red (ER); and (**b**) carminic acid (CA).

Ethyl red (ER) with a purity of greater than 98.0% was obtained from TCI (TCI (Shanghai) Development Co., Ltd., Shanghai, China), which was used without further purification. Carminic acid (CA) was obtained from Dr. Ehrenstorfer GmbH (Germany) and used without further purification. The chemical structures of the two dyes are shown in Figure 1. The ethanol and tetrahydrofuran (THF) solvents were used as received from Tianjin Kemiou Chemical Reagent Co., Ltd. (Tianjin, China).

UV-Vis spectra were measured with a TU-1900 spectrometer (Beijing Purkinje General Instrument Co., Ltd., Beijing, China). FT-IR spectra were measured with a FT-IR 360 spectrometer (Nicolet, Madison, WI, USA). Cyclic voltammetry experiments were performed using CH Instruments CHI615E Electrochemical Workstation (Shanghai Chenhua Instrument Co., Ltd., Shanghai, China). The redox potentials of the dyes were measured in a tetrahydrofuran (THF) solution, using 0.1 M KNO_3_ as the supporting electrolyte. The scan range was between −1000 mV and +1000 mV, and the initial scan potential was −1000 mV, at a scan rate of 50 mV/s, with a three-electrode system consisting of a glassy carbon working electrode, a platinum counter electrode, and an Ag/AgCl reference electrode.

The fabricated DSSC structure mainly includes dyes, an electrode, and an electrolyte, and the details of manufacturing process are as follows. (a) The TiO_2_ electrode was prepared by adding 10 mL isopropyl tianate to water and keeping hydrolysis for 3 h, and then adding nitric acid and acetate to the solution and placing it in an environment of 80 °C; the mixed solution was stirred until it became a transparent blue solution; later, the hydrothermal reaction was executed at 200 °C for 12 h. After cooling, spin steaming, and centrifuging, terpineol ethyl and cellulose were added to the ball grinder; the paste was prepared completely via ball mill, rotary steam, and three roll; (b) The screen printing technology was adopted to print the TiO_2_ paste to the clean surface of conductive glass, and the active area of the cell was 0.16 cm^2^; after ethanol bathing and drying, the anode electrodes were sintered and then treated in a TiCl_4_ solution. After this, the anode electrodes were sintered again. By the measurement of a surface roughness tester (TIME3100, Tiancheng Technology Co. Ltd., Beijing, China), the thickness of the TiO_2_ anode layer was about 16–18 μm. In later processing, the anode electrodes were immediately removed after naturally cooling to 80 °C, and the anode electrodes were soaked in the natural dye without light for 24 h; (c) The anode electrode and the platinum plating counter electrode were fitted together into the cell; in the middle of the two electrodes, the electrolyte solution (including 0.5 mol/L LiI, 0.05 mol/L I2 TBP, GUSCN) was added. The photoelectric conversion efficiency measurements of the DSSCs were carried out using a solar simulation instrument (Pecell-15, Peccell Technologies, Inc., Yokohama, Japan), and light intensity was tinkered up via a reference standard Si-solar solar cell at 1 sun light intensity of 100 mW·cm^−2^. Moreover, to determine the adsorbed amount of the two dyes on TiO_2_ thin films, adsorption–desorption experiments of the two dyes were performed according to previous research works [40,41,42]. The measurement of the incident photon-to-current conversion efficiency (IPCE) was performed by a Hypermonolight (SM-25, Jasco Co. Ltd., Tokyo, Japan).

### 2.2. Theory

In theory, the ground state structures of ethyl red (ER) and carminic acid (CA) were optimized via DFT [43,44], using B3LYP [45,46,47] functional with a 6-31G(d) basis set. On this basis, FT-IR spectra, the total static first hyperpolarizability and the frontier molecular orbital energies of the dyes in vacuum and solvent were obtained. The total static first hyperpolarizability can be written as follows [48]:

(1) $$\beta_{tot}=\sqrt{\beta_{x}^{2}+\beta_{y}^{2}+\beta_{z}^{2}},$$

Individual static component in the above equation is calculated from

(2) $$\beta_{i}=\beta_{iii}+\frac{1}{3}\sum_{i\neq j}\left(\beta_{ijj}+\beta_{jij}+\beta_{jji}\right),$$

where $\beta_{ijk}$ (*i*, *j*, *k* = *x*, *y*, *z*) are tenser components of the total static first hyperpolarizability. Due to Kleinman symmetry, one finally obtains the following equation:

(3) βtot=[(βxxx+βxyy+βxzz)2+(βyyy+βyzz+βyxx)2+(βzzz+βzxx+βzyy)2]12.

Moreover, the transition properties of the dyes in solvents were calculated using the TD-DFT [49,50] method based on the optimization of the ground state structures in the solvent. In order to select the appropriate functional and basis set to calculate the transition characteristics of dyes, the absorption properties of ethyl red and carminic acid in solvents were performed with different functional and basis set. The calculated results are listed in Table S1 (see Supplementary Materials Table S1). Finally, according to the simulated results, we selected PBEPBE/6-311++G(d,p) and MPW1PW91/6-311++G(d,p) to calculate the transition properties of ethyl red (ER) and carminic acid (CA), respectively. All calculations in solvents adopted the Conductor-like PCM (C-PCM) model [51] in this work. In addition, the ground state and excited state properties of dye/Ti(OH)_3_H_2_O complexes were calculated with the model proposed by Peng and co-workers [52], and other researchers have demonstrated the reliability of this simple model being used to analyze the properties of dyes [53,54]. The three-dimensional (3D) real-space analysis method [55,56] was used to describe the charge transfer process in dye/TiO_2_ complexes. Meanwhile, the diagrams of the density of state (DOS) and partial density of state (PDOS) of the two dyes adsorbed on TiO_2_ were presented using the Multiwfn 3.3.7 program (Beijing Quanton Technology Co. Ltd., Beijing, China) [57]. All calculations were performed using Gaussian 09 package [58].

## 3. Results and Discussion

### 3.1. Optical Characteristics of the Dyes

**Figure 2:**
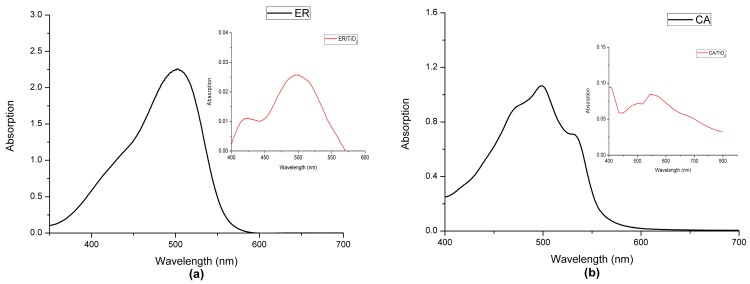
Absorption spectra of (**a**) ER and ER/TiO_2_; and (**b**) CA and CA/TiO_2_ in experiment.

The experimental absorption spectra of the two dyes in ethanol and the dyes with TiO_2_ are presented in Figure 2a,b, and the corresponding absorption peaks are listed in Table 1. As shown in Figure 2a,b, ethyl red (ER) and carminic acid (CA) all show the relatively strong absorption at 400–550 nm, at which the maximum absorption peaks of ethyl red and carminic acid are located on 502.50 and 499.00 nm, respectively (see Table 1). It is worth noting that the absorption ranges of the two dyes mainly distribute at the visible region, which is conducive to the effective use of solar energy. In addition, it can be found from Table 1 that the absorption peak of ethyl red with TiO_2_ has little change compared with that of the isolated dye. However, for carminic acid with TiO_2_, the absorption peak has a red shift of 32 nm in comparison with that of the isolated dye.

In order to deeply investigate the excited state properties of the dyes, the transition properties of the two dyes were calculated in ethanol using the TD-DFT method, with PBEPBE/6-311++G(d,p) and MPW1PW91/6-311++G(d,p) for ethyl red and carminic acid, respectively, based on the optimized ground state structures. The calculated absorption peaks and corresponding oscillator strengths are listed in Table 1. It can be seen from Table 1 that, for the dye ethyl red, the excited state S2 corresponds to the first strongest absorption 483.18 nm (*f* = 0.6403), which is composed by electrons transferring from H→L. The excited state S1 corresponds to the second strongest absorption 516.42 nm (*f* = 0.2843), which comes from the electrons transferring from H-1→L. The excited states S3, S4, S5, and S6 originate from the electrons transferring from H→L+1, H-2→L, H-1→L, and H-4→L, respectively, in spite of their negligible absorption intensity. Meanwhile, it can be seen from Table 1 that, for the transition properties of carminic acid, the excited state S1 corresponds to the first strongest absorption 485.78 nm (*f* = 0.2331), which originates from the electron transition from H→L. The excited state S4 corresponds to the second strongest absorption 352.82 nm (*f* = 0.1365), which has the contribution of electrons transition from H-2→L. Moreover, the excited states S2, S3, S5, and S6 correspond to the electrons transition from H-1→L, H-4→L, H-9→L, and H→L+1, respectively, ignoring their weak absorption.

**Figure 3:**
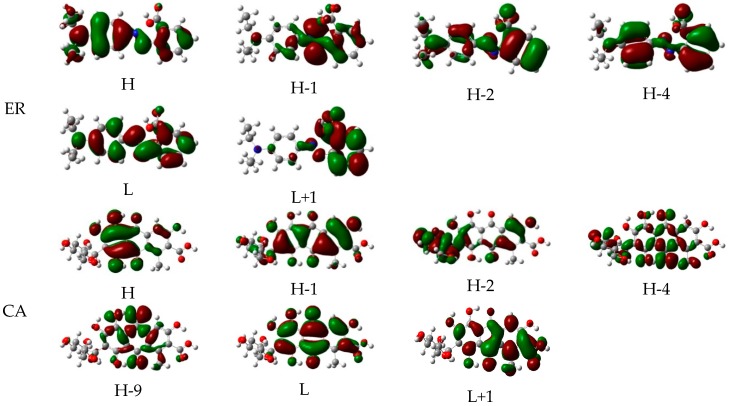
Selected frontier molecular orbitals of ethyl red (ER) and carminic acid (CA) in ethanol.

Moreover, the selected frontier molecular orbitals of the two dyes are shown in Figure 3, which are used to explain the electronic excitation and transition characteristics of dyes. It can be found from Figure 3 that, for ethyl red, the molecular orbitals of the HOMO and HOMO-2 spread over the entire molecule, whereas the molecular orbital of HOMO-1 mainly distributes at the 2-diazenylbenzoic acid unit. The molecular orbital of HOMO-4 mainly localizes on the benzene ring and 2-diazenylbenzoic acid unit. In addition, the molecular orbital of LUMO is mainly located on the benzene ring and the 2-diazenylbenzoic acid unit, whereas the molecular orbital of LUMO+1 is mainly distributed at the 2-diazenylbenzoic acid unit. From the distribution of the above molecular orbitals, electron injection is most likely to occur from the diethylamine unit to the 2-diazenylbenzoic acid unit. Simultaneously, for carminic acid, the molecular orbitals of HOMO and HOMO-9 are located on the 1,2,4-trihydroxy-5-methylanthracene-9,10-dione unit, and that of HOMO-1 mainly spreads over the molecular backbone. At the HOMO-2 level, the molecular orbital is located on the (2R,3S,4R,5S)-2-(hydroxymethyl)-tetrahydro-2H-pyran-3,4,5-triol unit and the toluene of the 1,2,4-trihydroxy-5-methylanthracene-9,10-dione unit. The molecular orbitals of HOMO-3 and HOMO-4 spread almost over the entire molecule. Meanwhile, the molecular orbitals of LUMO and LUMO+1 are located on the 1,2,4-trihydroxy-5-methylanthracene-9,10-dione unit.

### 3.2. FT-IR Spectra

**Figure 4:**
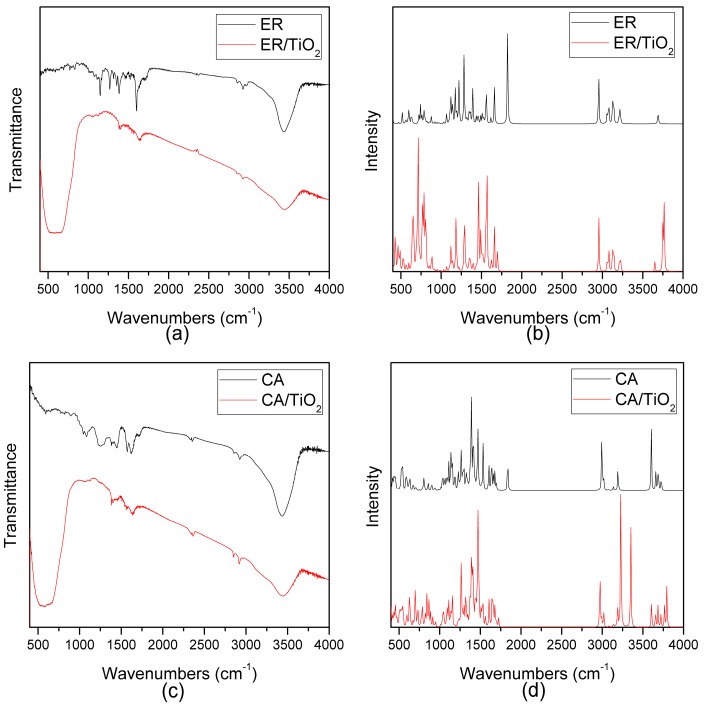
(**a**) Experimental FT-IR for ER and ER/TiO_2_; (**b**) simulated FT-IR for ER and ER/TiO_2_; (**c**) experimental FT-IR for CA and CA/TiO_2_; (**d**) simulated FT-IR for CA and CA/TiO_2_.

The experimental and simulated FT-IR spectra of the two dyes and dye/TiO_2_ complexes in the range of 400–4000 cm^−1^ are shown in Figure 4. It can be seen from Figure 4a,b that, for ethyl red, the strong IR intensity mainly distributes in the region of 1000–2000 cm^−1^ and 3000–4000 cm^−1^, including the characteristic peaks for ethyl red 1148.33 cm^−1^, 1267.70 cm^−1^, 1354.54 cm^−1^, 1383.86 cm^−1^, 1600.81 cm^−1^, and 3435.10 cm^−1^. The vibration analysis corresponding to the characteristic peaks of the two dyes are displayed in Figures S1 and S2 (see Supplementary Materials Figures S1 and S2). As shown in Figure S1, the characteristic peaks at 1148.33 cm^−1^ and 1600.81 cm^−1^ mainlyoriginate from the vibration of C–H located on the benzene ring of the molecular middle. The characteristic peak at 1267.70 cm^−1^ mainly comes from the vibration of C–H located on the benzene ring of the molecular middle and the 2-diazenylbenzoic acid unit. The characteristic peaks 1354.54 cm^−1^ and 1383.86 cm^−1^ mainly derive from the vibration of C–H on the diethylamine unit and the 2-diazenylbenzoic acid unit, respectively. Moreover, the characteristic peak at 3435.10 cm^−1^ stems from the stretching vibration of O–H on the carboxyl unit. As can be seen from Figure 4a, compared with the FT-IR spectrum of isolated dye ethyl red, the peak located at about 3500.00 cm^−1^ in the FT-IR of dye/TiO_2_ complex become weaker, indicating that the O–H bond on the carboxyl unit of ethyl red ruptures. In response to this, the characteristic peak appears in the 400–700 cm^−1^ region (see Figure 4a), corresponding to the stretching vibration of the Ti–O bond, which means that the Ti–O bond is formed; the dye had adsorbed on the TiO_2_. These features can also be supported by the results of theoretical simulation (see Figure 4b): there is a peak at 3689.71 cm^−1^ in the FT-IR of the isolated dye, originating from the stretching vibration of O–H bond on the carboxyl unit, which disappeared in the FT-IR of the dye/TiO_2_ complex; a peak at 487.69 cm^−1^ appears in the FT-IR of the dye/TiO_2_ complex, which corresponds to the stretching vibration of the Ti–O bond. From the experimental and theoretical results, it can be seen that the dye ethyl red had adsorbed on the TiO_2_ film.

Meanwhile, it can be found from Figure 4c,d that, for carminic acid, the strong IR intensity mainly distributes in the region of 1000–2000 cm^−1^ and 3000–4000 cm^−1^, i.e., 1081.97 cm^−1^, 1249.99 cm^−1^, 1446.34 cm^−1^, 1574.08 cm^−1^, 1621.35 cm^−1^, and 3439.51 cm^−1^. As shown in Figure S2, the characteristic peaks at 1081.97 cm^−1^, 1249.99 cm^−1^, and 1446.34 cm^−1^ are mainly produced by the vibrations of C–H and O–H on the (2R,3S,4R,5S)-2-(hydroxymethyl)-tetrahydro-2H-pyran-3,4,5-triol. Moreover, the characteristic peaks at 1574.08 cm^−1^ and 1621.35 cm^−1^ are due to the vibration of C–H and O–H on the 1,2,4-trihydroxy-5-methylanthracene-9,10-dione unit. In addition, the characteristic peak at 3439.51 cm^−1^ comes from the stretching vibration of O–H on the 1,2,4-trihydroxy-5-methylanthracene-9,10-dione unit. Furthermore, the peak located at about 3500.00 cm^−1^ in the FT-IR of the dye/TiO_2_ complex becomes weaker compared with that of the isolated dye, implying that the O–H bond on the carboxyl unit of carminic acid ruptures (see Figure 4c). Similarly, the peak at 3602.72 cm^-1^ in the simulated FT-IR spectrum of the isolated dye, which originates from the stretching vibration of O–H on the carboxyl unit of carminic acid, disappears from that of the dye/TiO_2_ complex. Moreover, the characteristic peak, which corresponds to the stretching vibration of the Ti–O bond, appears in the 400–700 cm^−1^ region (see Figure 4c); it also can be found from the simulated results that the peak at 729.47 cm^−1^ appears in the FT-IR of the dye/TiO_2_ complex, which corresponds to the stretching vibration of the Ti–O bond. It also shows that the dye is adsorbed on the TiO_2_ film effectively.

### 3.3. Photovoltaic Properties of Fabricated DSSCs

The overall photoelectric conversion efficiency, η, isdefined as follows [59]:

(4) $$\eta =\frac{J_{\mathrm{SC}}\;\times \;V_{\mathrm{OC}}\times ff}{P},$$

where $J_{\mathrm{sc}}$ is short-circuit current density, $V_{\mathrm{oc}}$ is open circuit voltage, ff is the fill factor, and P is the intensity of the incident light.

The fill factor (ff) is defined as the ratio of the maximum power obtained from the DSSC and the theoretical maximum power, which is formulated as

(5) $$ff=\frac{I_{m}\times V_{m}}{J_{\mathrm{SC}}\;\times \;V_{\mathrm{OC}}},$$

where $I_{m}$ and $V_{m}$ are current and voltage related to the maximum power, respectively.

**Figure 5:**
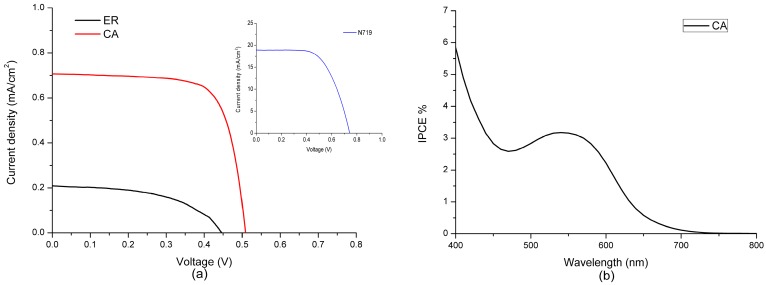
(**a**) Current–voltage curves of DSSCs sensitized with ethyl red (ER), carminic acid (CA), and N719, respectively; (**b**) IPCE for two DSSCs based on the two dyes.

Here, the photovoltaic characteristics of the DSSCs sensitized with the two dyes and referenced N719 are listed in Table 2, consisting of open circuit voltage ($V_{\mathrm{oc}}$), short-circuit current density ($J_{\mathrm{sc}})$, fill factor (ff) and photoelectric conversion efficiency (η %). In addition, the current–voltage characteristics of the DSSCs sensitized with the two dyes and N719 are shown in Figure 5a. As listed in Table 2, compared with the photovoltaic characteristics of the DSSC sensitized with ethyl red, the DSSC sensitized with carminic acid shows a higher photoelectric conversion efficiency of 0.30%, with a higher open circuit voltage of 0.53 V, a short-circuit current density of 0.66 mA cm^−2^ and a fill factor of 0.84. It should be noted that the photoelectric conversion efficiency of carminic acid is six times that of ethyl red. The statistical amounts of the two dyes adsorbed on the TiO_2_ photoanode, tested via dye desorption, are also listed in Table 2. It can be found that the amount of carminic acid adsorbed on the TiO_2_ photoanodeis higher than that of the ethyl acid adsorbed on the TiO_2_ photoanode, indicating that the carminic acid dye is more easily adsorbed on the TiO_2_ photoanode. According to previous research works by Cojocaru et al. [42] and Jena et al. [60], the greater the amount of dye adsorbed on the TiO_2_ photoanode is, the higher the short-circuit current density is, thereby improving the photoelectric conversion efficiency, which is in agreement with the experiment.

The incident photo-to-current conversion efficiency (IPCE) measurement contributes to the further understanding for the photovoltaic characteristics of DSSC. By measuring the IPCE spectra of the DSSCs sensitized with the two dyes in the visible light region, we found that the DSSCs sensitized with ethyl red were too weak in the visible light region; therefore, the IPCE spectrum of the DSSC sensitized with carminic acid is mainly discussed in this paper. The IPCE spectrum of the DSSC sensitized with carminic acid is shown in Figure 5b. It can be found from Figure 5b that, for the dye carminic acid, the IPCE spectrum of the dye has a peak when the wavelength of incident light is at 500–600 nm, which may cause a greater short-circuit current density for carminic acid.

### 3.4. Electrochemical Characteristic

**Figure 6:**
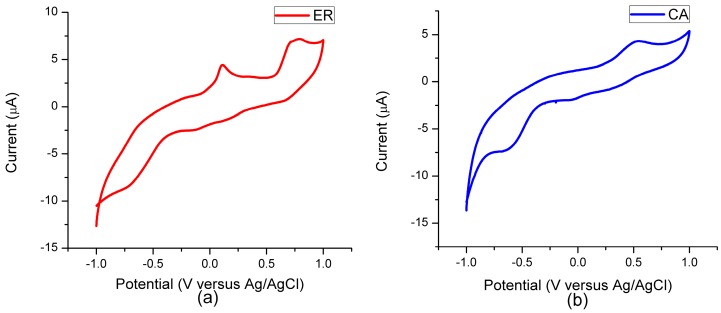
Cyclic voltammograms of (**a**) ethyl red (ER); and (**b**) carminic acid (CA).

The investigation of electrochemical properties can reflect the characteristics of electron transition from an excited state of dye to the conduction band of the semiconductor and the ability of dye regeneration. The electrochemical characteristics of ethyl red and carminic acid were investigated by cyclic voltammetry measurements in a tetrahydrofuran (THF) solvent using KNO_3_ as a supporting electrolyte, and the cyclic voltammograms of the two dyes are shown in Figure 6. Because the cyclic voltammetry measurements of the two dyes in ethanol solvent were imperfect, we replaced the ethanol solvent by the tetrahydrofuran solvent. After calculation, the onset oxidation potentials of the two dyes were 0.06 V for ethyl red and 0.28 V for carminic acid, respectively, which is obtained by the intersection of two tangent lines for the rising current curve and the starting current curve, respectively. The HOMO energy corresponds to the onset oxidation potential of the dye, when an Ag/AgCl electrode was adopted as the reference electrode, and the HOMO energy can be calculated according to the following formula [61]: $\mathrm{HOMO}=-e\left(E_{\mathrm{OX}}+4.40\right)\;\left(\mathrm{eV}\right)$, where $E_{\mathrm{OX}}$ represents the onset oxidation potential of dye. Therefore, the HOMO energies of the two dyes are −4.46 eV for ethyl red and −4.68 eV for carminic acid, respectively. In general, the electron donor with strong donating ability will shift the HOMO level more negative [62]. In view of this, the dye carminic acid has the greater donating electron ability.

**Figure 7:**
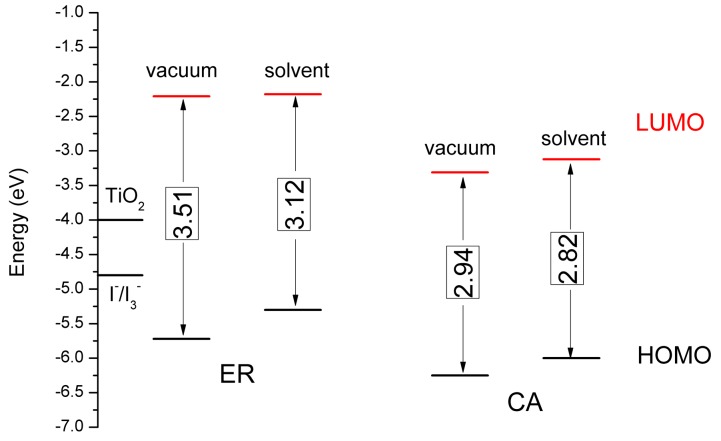
Calculated HOMOs, LUMOs and energy gaps ($\Delta_{H-L}$) of ethyl red (ER) and carminic acid (CA) in vacuum and solvent.

In order to compare the experimental values, the calculated HOMOs, LUMOs, and energy gaps ($\Delta_{\mathrm{Hn}\;}$) of the two dyes in vacuum and solvent were obtained based on the optimized molecular structures. The results are presented in Figure 7, and the data are listed in Table S2 (see Supplementary Materials Table S2). If the DSSC intends to have high photoelectric conversion efficiency, the dye will be sure to have suitable HOMO and LUMO energies. The LUMO energy should be higher than the conduction band edge of TiO_2_ (about −4.0 eV) to ensure that the electrons in the dye excited state can be injected into the conduction band of the semiconductor, and the HOMO energy would be lower than that of I^−^/I_3_^−^ (about −4.8 eV) to ensure that the dye in the oxidation state can be deoxidated by the electrolyte [63,64]. It can be found from Figure 7 that the HOMOs of the two dyes in solvent are higher than that in vacuum, and the HOMOs of the two dyes in solvent are lower than the energy of I^−^/I_3_^−^ (−5.30 eV for ethyl red and −6.00 eV for carminic acid, respectively, see Table S2), implying that the two dyes in the oxidation state can be deoxidated by the electrolyte. Moreover, it can be seen from Figure 7 that the LUMOs of the two dyes in solvent are higher than that in vacuum, and the LUMOs of the two dyes in solvent are higher than the conduction band edge of TiO_2_ (−2.18 eV for ethyl red and −3.12 eV for carminic acid, respectively, see Table S2), indicating that the electrons in the excited state of the two dyes can be effectively injected into the conduction band of the semiconductor. In addition, carminic acid has a lower HOMO in vacuum compared with that of ethyl red, which is similar to the phenomenon in solvent.

Moreover, it can be seen from Figure 7 that the energy gaps of the two dyes in solvent (3.12 eV for ethyl red and 2.82 eV for carminic acid, respectively) are smaller than that in vacuum (3.51 eV for ethyl red and 2.94 eV for carminic acid, respectively). It is worth noting that the energy gaps of carminic acid in vacuum and solvent are all narrower than that of ethyl red. Therefore, carminic acid will show the red-shifted absorption spectrum, which may be beneficial for obtaining higher short-circuit current density and photoelectric conversion efficiency [65].

### 3.5. Theoretical Analysis for $J_{\mathrm{sc}}$ and $V_{\mathrm{oc}}$

From Equation (4), it can be found that the photoelectric conversion efficiency of DSSC is mainly determined by the short-circuit current density ($J_{\mathrm{sc}}$), open circuit voltage ($V_{\mathrm{oc}}$), and fill factor (ff). It is known that theshort-circuit current density ($J_{\mathrm{sc}}$) is determined by the light harvesting efficiency (LHE) of dye, the injection efficiency of the electrons in the excited state ($\Phi_{\mathrm{inj}}$), the charge collection efficiency of TiO_2_ electrode ($\eta_{\mathrm{coll}}$), and the regeneration efficiency of the dye ($\eta_{\mathrm{reg}}$) [59,66], which is formulated as

(6) $$J_{\mathrm{sc}}=e\int \mathrm{LHE}\left(\lambda \right)\Phi_{\mathrm{inj}}\eta_{\mathrm{coll}}\eta_{\mathrm{reg}}I_{s}\left(\lambda \right)d\lambda .$$

For a given DSSC, it can be interpreted that the charge collection efficiency ($\eta_{\mathrm{coll}}$) has little difference because of the same semiconductor electrode (usually TiO_2_) [54]. Hereto, the short-circuit current density ($J_{\mathrm{sc}}$) is determined by three parameters: the light harvesting efficiency (LHE), the injection efficiency of the electrons in the excited state ($\Phi_{\mathrm{inj}}$), and the regeneration efficiency of the dye ($\eta_{\mathrm{reg}}$), in which the light harvesting efficiency (LHE) can be described by [67,68]:

(7) $$\mathrm{LHE}=1-10^{-f},$$

where *f* is the calculated oscillator strength. According to Equation (7), the calculated LHE of the two dyes are 0.7711 and 0.3840 for ethyl red and carminic acid, respectively. Although ethyl red presents a higher LHE compared with that of carminic acid, we cannot arbitrarily think that ethyl red will have a higher short-circuit current density due to the LHE of the two dyes obtained by different calculation methods.

In addition, the effect of injection efficiency of the electrons in an excited state ($\Phi_{\mathrm{inj}}$) on the short-circuit current density ($J_{\mathrm{sc}}$) was investigated. The $\Phi_{\mathrm{inj}}$ is related to the driving force of the electron injection ($\Delta G^{\mathrm{inject}}$). According to Preat’s method [69], the $\Delta G^{\mathrm{inject}}$ can be expressed as

(8) $$\Delta G^{\mathrm{inject}}=E_{\mathrm{OX}}^{\mathrm{dye}*}-E_{\mathrm{CB}},$$

where $E_{\mathrm{OX}}^{\mathrm{dye}*}$ is the excited state oxidation potential, and $E_{\mathrm{CB}}$ is the reduction potential of the conduction band of the semiconductor. In general, the anatase TiO_2_ is used as the electrode for DSSCs, and the reported $E_{\mathrm{CB}}$ for anatase TiO_2_ (about 4.0 eV) [70] was adopted in this work as reference value. Moreover, the $E_{\mathrm{OX}}^{\mathrm{dye}*}$ can be calculated by the following equation [71]:

(9) $$E_{\mathrm{OX}}^{\mathrm{dye}*}=E_{\mathrm{OX}}^{\mathrm{dye}}-\lambda_{\max},$$

where $E_{\mathrm{OX}}^{\mathrm{dye}}$ is the ground state oxidation potential, and $\lambda_{\max}$ is the maximum absorption. The calculated $E_{\mathrm{OX}}^{\mathrm{dye}}$, $E_{\mathrm{OX}}^{\mathrm{dye}*}$, and $\Delta G^{\mathrm{inject}}$ are listed in Table 3, and it can be found that the $\Delta G^{\mathrm{inject}}$ of the two dyes are 1.27 eV and 0.55 eV for ethyl red and carminic acid, respectively. In terms of the research work of Islam et al. [72], the injection efficiency of the electrons in the excited state ($\Phi_{\mathrm{inj}}$) tends to 1 when the $\Delta G^{\mathrm{inject}}$ is greater than 0.2 eV. Therefore, it can be consideredthat the two dyes have the same $\Phi_{\mathrm{inj}}$.

Meanwhile, the regeneration efficiency of the dye ($\eta_{\mathrm{reg}}$) is determined by the driving force of the dye regeneration ($\Delta G^{\mathrm{regen}}$). The $\Delta G^{\mathrm{regen}}$ can be described by [73]

(10) $$\Delta G^{\mathrm{regen}}=E_{\mathrm{OX}}^{\mathrm{dye}}-E_{\mathrm{redox}}^{\mathrm{electrolyte}},$$

where $E_{\mathrm{redox}}^{\mathrm{electrolyte}}$ is the redox potential of the electrolyte. In this work, the $E_{\mathrm{redox}}^{\mathrm{electrolyte}}$ of redox couple iodide/triiodide (about 4.80 eV) [63,64] was adopted to evaluate the $\Delta G^{\mathrm{regen}}$. The calculated $\Delta G^{\mathrm{regen}}$ are listed in Table 3, and it can be seen that carminic acid has the higher $\Delta G^{\mathrm{regen}}$ (1.4 eV) compared with that of ethyl red (0.7 eV), which would result in carminic acid’s higher short-circuit current density. Through the analysis of the light harvesting efficiency (LHE) of the dye, the injection efficiency of the electrons in the excited state ($\Phi_{\mathrm{inj}}$), and the regeneration efficiency of the dye ($\eta_{\mathrm{reg}}$), the improved $\eta_{\mathrm{reg}}$ of carminic acid is caused by the fact that larger $\Delta G^{\mathrm{regen}}$ is favorable to the greater short-circuit current density, which is in agreement with the experimental value.

The open circuit voltage ($V_{oc}$) is the difference between the quasi-Fermi level of the electron in the TiO_2_ conduction band and the redox potential of electrolyte, which can be expressed as [74]

(11) $$V_{\mathrm{OC}}=\frac{E_{\mathrm{CB}}+\Delta E_{\mathrm{CB}}}{q}+\frac{\kappa_{b}T}{q}\ln\left(\frac{n_{c}}{N_{\mathrm{CB}}}\right)-\frac{E_{\mathrm{redox}}}{q},$$

where *q* is the unit charge, $E_{\mathrm{CB}}$ represents the conduction band edge of the semiconductor, $\kappa_{b}T$ is the thermal energy, $n_{c}$ is the number of electrons in the conduction band, $N_{\mathrm{CB}}$ represents the density of accessible states in the conduction band, and $E_{\mathrm{redox}}$ stands for the electrolyte Fermi level. $\Delta E_{\mathrm{CB}}$ represents the shift of $E_{\mathrm{CB}}$ when the dyes are adsorbed on the semiconductor substrate and can be described by [75,76]

(12) $$\Delta E_{\mathrm{CB}}=\frac{-q\mu_{\mathrm{normal}}\gamma }{\varepsilon_{0}\varepsilon },$$

where γ is the concentration of the dyes absorbed on the surface of the semiconductor, $\mu_{\mathrm{normal}}$ is the dipole moment component of the dye molecules perpendicular to the surface of TiO_2_, and $\varepsilon_{0}$ and ε present the dielectric constant in vacuum and the organic monolayer, respectively. According to Equations (11) and (12), the $\mu_{\mathrm{normal}}$ and $\Delta E_{\mathrm{CB}}$ have a close relationship with $V_{\mathrm{oc}}$. Obviously, the dyes with larger $\mu_{\mathrm{normal}}$ and $\Delta E_{\mathrm{CB}}$ will generate a larger $V_{\mathrm{oc}}$. The calculated $\mu_{\mathrm{normal}}$ of the two dyes are listed in Table 3, in which it can be seen that carminic acid has the larger $\mu_{\mathrm{normal}}$ (7.77 D) compared with that of ethyl red (2.20 D). In addition, the density of the state and the partial density of state (PDOS) of the two dyes adsorbed on TiO_2_ are presented in Figures S3 and S4 (see Supplementary Materials Figures S3 and S4). As shown in Figures S3 and S4, carminic acid has a $\Delta E_{\mathrm{CB}}$ of 0.410 eV, larger than that of ethyl red (0.337 eV). The above results indicate that carminic acid would have a greater $V_{\mathrm{oc}}$ due to the larger $\mu_{\mathrm{normal}}$ and $\Delta E_{\mathrm{CB}}$, which is in agreement with the experimental results.

### 3.6. Excited State Lifetime (τ)

Excited state lifetime is one of the important parameters to study the efficiency of charge transfer [77]. The longer the excited state lifetime is, the longer the time of dyes maintains in the cationic form is, which is more conducive to the charge transfer [65,77]. The excited state lifetime of the dye can be evaluated via the following equation: $\tau =1.499∕fE^{2}$, where *E* is the excitation energy of the different electronic states (cm^−1^) and *f* is the oscillator strength corresponding to the electronic state [65]. The calculated excited state lifetimes of the two dyes are listed in Table 3. As listed in Table 3, the excited state lifetimes of ethyl red and carminic acid are 14.05 ns and 15.20 ns, respectively. The results imply that carminic acid remains stable in the cationic state for a longer time, which engenders a higher charge transfer efficiency and enhanced efficiency of the DSSC. This result is in agreement with the experimental results.

### 3.7. Total Static First Hyperpolarizability

Due to the important application of hyperpolarizability in the research of molecular nonlinear optical (NLO) properties, the first hyperpolarizabilities of the two dyes were investigated in vacuum and solvent, and the results are listed in Table 4. It can be seen from Table 4 that the first hyperpolarizabilities of the two dyes in vacuum and solvent are all in this order: ER > CA. It is worth noticing that the components of the first hyperpolarizabilities of the two dyes are all mainly along the *x*-axis, which is also the direction of the charge transfer. Although the first hyperpolarizability of ethyl red is larger than that of carminic acid, the photoelectric conversion efficiency of DSSC based on ethyl red presents a lower value due to the non-planar structure between the bridge and acceptor, which restrained the electron transferring from donor to acceptor, thereby affecting the effective electron injection from the dye molecule to the conduction band of the semiconductor.

### 3.8. Properties of Dye/TiO_2_ Complexes

**Figure 8:**
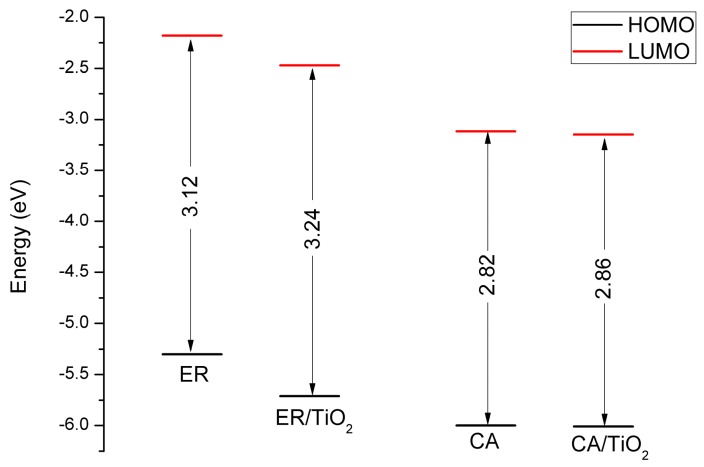
Frontier molecular orbital levels of the isolated dyes and the dye/TiO_2_ complexes in ethanol.

In order to simulate the photoelectrical properties of DSSC more realistically, the electronic and optical characteristics of the dyes adsorbed on TiO_2_ were investigated using the DFT and TDDFT methods. The calculated frontier molecular orbital levels of the isolated dyes and the dye/TiO_2_ complexes in ethanol are presented in Figure 8, and the detailed data are listed in Table S3 (see Supplementary Materials Table S3). As shown in Figure 8, the HOMO and LUMO energies of the dye ethyl red adsorbed on TiO_2_ are lower than that of the isolated dye, and the energy gap increases by 0.12 eV compared with that of the isolated dye. Meanwhile, the HOMO and LUMO energies of the dye carminic acid adsorbed on TiO_2_ show almost no change compared with that of the isolated dye, which is also the case for the energy gap.

**Figure 9:**
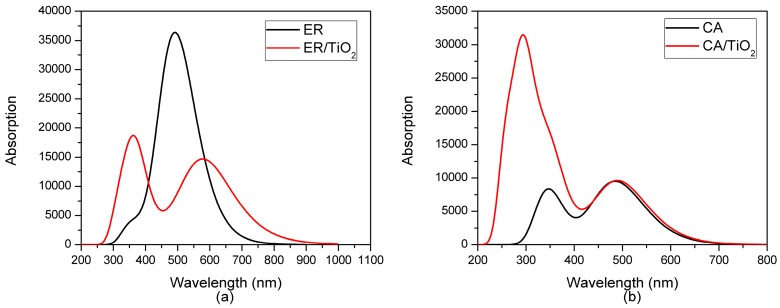
Simulated UV-Vis spectra of (**a**) ER and ER/TiO_2_; and (**b**) CA and CA/TiO_2_ in ethanol.

The transition properties of the dye/TiO_2_ complexes in ethanol were calculated at the PBEPBE/6-311++G(d,p) and MPW1PW91/6-311++G(d,p) levels for ER/TiO_2_ and CA/TiO_2_ complexes, respectively. The selected absorption peaks and corresponding oscillator strengths of the two dye/TiO_2_ complexes are listed in Table 5, and the complete data for the first 30 excited states are listed in Tables S4 and S5 (see Supplementary Materials Tables S4 and S5). The simulated UV-Vis spectra of the isolated dyes and dye/TiO_2_ complexes in ethanol are shown in Figure 9. Previous works have shown that the photo-injection mechanism can be divided into two types depending on the photo-injection mechanism from the dye to the semiconductor [78,79]: the first mechanism Type I (indirect) contains the photo-excitation to a dye excited state, from which the electrons are transferred to the semiconductor; the second mechanism Type II (direct) involves the electrons injecting from the ground state of the dye to the conduction band of the semiconductor. According to previous works [80,81], the photo-injection mechanism can be investigated via comparing the UV-Vis spectrum of the isolated dye with that of the dye/semiconductor complex. Compared with the UV-Vis spectrum of the isolated dye, the emergence of a new band in the spectrum of the dye/semiconductor complex means that the complex undergoes a Type II (direct) mechanism. In addition, if the UV-Vis spectrum of the dye/semiconductor complex has no new band compared with that of the isolated dye, the complex exhibits a Type I (indirect) mechanism. As shown in Figure 9, the absorption spectrum of the ER/TiO_2_ complex has a new band compared with that of the isolated dye, implying that the ER/TiO_2_ complex exhibits a Type II (direct) injection route. However, for the CA/TiO_2_ complex, the absorption spectrum presents no new band compared with that of the isolated dye, indicating that the complex shows a Type I (indirect) injection route.

**Figure 10:**
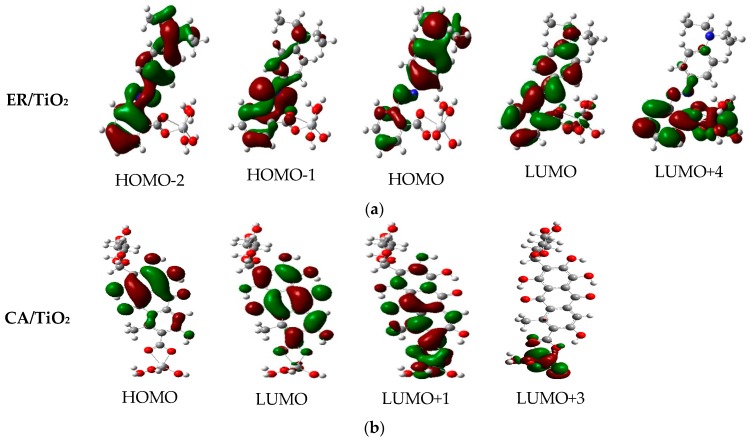
Selected frontier molecular orbitals of (**a**) ER/TiO_2_; and (**b**) CA/TiO_2_ complexes in ethanol.

As listed in Table 5, for the complex ethyl red anchored on TiO_2_ cluster, the excited state S1 corresponds to the strongest absorption (*f* = 0.3376), whose absorption peak is located on the point of 586.13 nm. This excited state derives from the electrons transition from HOMO to LUMO. Figure 10 presents the selected frontier molecular orbitals of the two dye/TiO_2_ complexes in ethanol. It can be found that, for the ER/TiO_2_ complex, the electron density of HOMO mainly distributes on the diethylamine and benzene ring units, and that of LUMO mainly distributes on the benzene ring, 2-diazenylbenzoic acid, and the TiO_2_ units. That is to say, the electron transition corresponding to the excited state S1 is in the direction from the dye molecule to the TiO_2_. The excited state S10 corresponds to the second stronger absorption state, which comes from the electron’s transition from HOMO-2 to LUMO with the oscillator strength *f* = 0.2385. Moreover, the electron transition corresponding to the excited state S10 is also in the direction from the dye molecule to the cluster (see Figure 10). The third stronger absorption state (S15) originates the electrons transition from HOMO-1 to LUMO+4, with the oscillator strength *f* = 0.0528. From Figure 10, the molecular orbital of LUMO+4 mainly distributes on the 2-diazenylbenzoic acid and TiO_2_ units. For those states, a change of electron density is propitious to the electron injection from the dye molecule to the semiconductor.

Simultaneously, it can be found from Table 5 that, for the complex carminic acid anchored on TiO_2_, the excited state S12 corresponds to the strongest absorption (*f* = 0.3310), which originates from the electrons transition from HOMO to LUMO+3. The excited state S1 corresponds to the second stronger absorption state, with the oscillator strength *f* = 0.2347, which originates from the electrons transition from HOMO to LUMO. The absorption peak corresponding to the excited state S1 has a red shift of 5.20 nm compared with the maximum absorption peak of the isolated dye. The third stronger absorption state (S5) originates the electrons transition from HOMO to LUMO+1, with oscillator strength *f* = 0.2007. Figure 10 shows that the electrons’ transitions corresponding to these three excited states in the direction from the dye to the TiO_2_.

**Figure 11:**
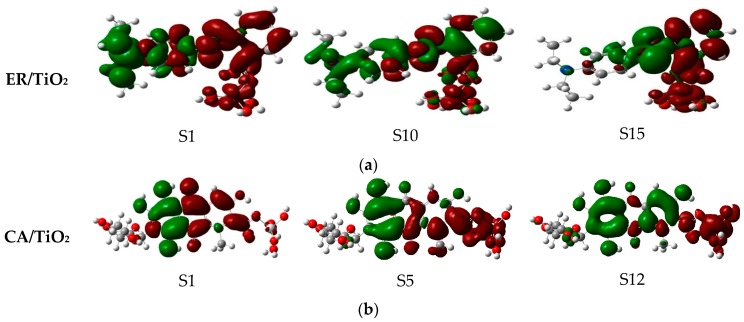
Visualization for the charge difference density (CDD) of the selected excited state for (**a**) ER/TiO_2_; and (**b**) CA/TiO_2_ complexes in ethanol.

In order to understand the charge-transfer properties of the excited state complexes, the charge difference density (CDD) of the two dyes adsorbed on the TiO_2_ complexes in ethanol are presented in Figure 11. As shown in Figure 11, for the ER/TiO_2_ complex, the excited states S1 and S15 are all total charge-transfer excited states, which implies the hole density and electron density are almost completely separated. For the excited state S10, some of the hole density and electron density are distributed on the dye molecule, and the rest of the electron density is distributed on the TiO_2_. For the CA/TiO_2_ complex, the hole density and electron density corresponding to the excited state S1 are almost completely separated on the dye molecule. For the excited states S5 and S12, some of the hole density and electron density are distributed on the dye molecule, and the rest of the electron density is located on the TiO_2_. In summary, the electrons in the six excited states are all in the direction from the dye to the TiO_2_.

### 3.9. Chemical Reactivity Parameters

On the base of optimized neutral and ionic structures, the following chemical reactivity parameters were calculated: electron affinity (*A*), ionization potential (*I*), chemical hardness (*h*), electrophilicity index (ω), electron donating power (ω^−^), and electron accepting power (ω^+^). The obtained results are listed in Table 6. Previous research has shown that the lower chemical hardness results in the lower resistance to intramolecular charge transfer [82,83], and the lower chemical hardness and higher electron accepting power lead to a better short-circuit current density, thereby generating excellent photoelectric conversion efficiency [84]. It can be found from Table 6 that the dye carminic acid possesses a lower chemical hardness (*h* = 1.21 eV) compared with that of ethyl red (*h* = 1.35 eV), indicating that carminic acid presents a lower resistance to intramolecular charge transfer. Moreover, carminic acid has a higher electron accepting power (ω^+^ = 6.50 eV) than does ethyl red (ω^+^ = 3.62 eV), which implies that carminic acid would show a higher ability to attract electrons by means of the acceptor moiety of the dye. Taking the above two parameters into account comprehensively, carminic acid would have a higher short-circuit current density, which is in agreement with the experimental results. By comparing the electrophilicity index of the two dyes, carminic acid has a higher electrophilicity index (8.63 eV) than that of ethyl red (5.35 eV), which indicates that carminic acid shows higher energetic stability by attracting the electrons from the environment [85]. On the part of electron donating power, the lower electron donating power leads to a greater ability of donating electrons [86]. Therefore, it can be seen from Table 6 that ethyl red exhibits lower electron donating power, implying that this dye has a greater ability of donating electrons. However, synthetically considering the chemical reactivity parameters of these two dyes, the photoelectrical properties of carminic acid would excel that of ethyl red.

## 4. Conclusions

The photoelectrical properties of the two dyes ethyl red and carminic acid as the sensitizers of dye-sensitized solar cells (DSSCs) were investigated in the above experiments. The frontier molecular orbital, the energy gaps, the absorption spectra, and the electronic properties of the two dyes before and after absorption on TiO_2_ were calculated via DFT and TD-DFT methods. The key parameters that were closely related to the short-circuit current density ($J_{\mathrm{sc}}$) and open circuit voltage ($V_{\mathrm{oc}}$), including the light harvesting efficiency (LHE), the excited state lifetime (τ), the driving force of electron injection ($\Delta G^{\mathrm{inject}}$) and dye regeneration ($\Delta G^{\mathrm{regen}}$), the total dipole moment ($\mu_{\mathrm{normal}}$), and the conduction band of the edge of the semiconductor ($\Delta E_{\mathrm{CB}}$) were investigated to reveal the intrinsic reason for the difference in the photoelectric conversion efficiency of the two dyes. The chemical reactivity parameters of the two dyes including electron affinity (*A*), ionization potential (*I*), chemical hardness (*h*), electrophilicity index (ω), electron donating power (ω^−^), and electron accepting power (ω^+^) were calculated. The following conclusions can be drawn from the calculated results: (a) A larger amount of dye adsorbed on a TiO_2_ photoanode, for carminic acid, leads to a higher short-circuit current density, thereby improving the photoelectric conversion efficiency of carminic acid; (b) It was found that the CA/TiO_2_ complexexhibits an indirect injection route since no new absorption bands appear in the absorption spectra of the complex; (c) Because of the larger $\Delta G^{\mathrm{regen}}$, excited state lifetime (τ), $\mu_{\mathrm{normal}}$, and $\Delta E_{\mathrm{CB}}$, carminic acid has a larger $J_{\mathrm{sc}}$ and $V_{\mathrm{oc}}$; (d) The lower chemical hardness (*h*) and higher electron accepting power (ω^+^) of carminic acid lead to a larger short-circuit current density, thereby generating excellent photoelectric conversion efficiency. It is expected that the molecule with a structure similar to carminic acid can possess photoelectric properties by molecular regulation.

## Figures and Tables

**Table 1:** Experimental absorption peaks and calculated transition properties of the two dyes in ethanol using the TD-DFT method, with PBEPBE/6-311++G(d,p) and MPW1PW91/6-311++G(d,p) for ethyl red (ER) and carminic acid (CA), respectively.

Dye	State	$\lambda_{\mathrm{abs}}$ (nm/eV)	Contribution MO	Strength *f*	$\mathrm{Exp}.^{\;a}$	Exp. ^b^
ER	S1	516.42/2.4009	(0.55904)H-1→L	0.2843	502.50	499.00
	S2	483.18/2.5660	(0.54705)H→L	0.6403		
	S3	396.25/3.1289	(0.66579)H→L+1	0.0578		
	S4	364.76/3.3991	(0.53490)H-2→L	0.0182		
	S5	353.83/3.5040	(0.54898)H-1→L	0.0242		
	S6	341.12/3.6346	(0.60041)H-4→L	0.0353		
CA	S1	485.78/2.5523	(0.70012)H→L	0.2331	499.00	531.00
	S2	394.10/3.1460	(0.67917)H-1→L	0.0111		
	S3	379.66/3.2657	(0.59339)H-4→L	0.0001		
	S4	352.82/3.5141	(0.61934)H-2→L	0.1365		
	S5	340.32/3.6432	(0.65265)H-9→L	0.0003		
	S6	333.64/3.7161	(0.55417)H→L+1	0.0785		

^a^ the measured absorption peaks in the experiments (concentration in 2 × 10^−4^ M); ^b^ the measured absorption peaks of dye/TiO_2_.

**Table 2:** Photovoltaic parameters of DSSCs based on ethyl red (ER), carminic acid (CA), and N719.

Dyes	*A* × 10^−8^ (mol·cm^−2^) ^a^	$V_{\mathrm{OC}}$ (V)	$J_{\mathrm{SC}}$ (mA·cm^−2^)	*ff*	η%
ethyl red (ER)	1.16	0.46	0.21	0.54	0.05
carminic acid (CA)	3.08	0.53	0.66	0.84	0.30
N719	–	0.74	18.91	0.62	8.74

^a^ Amount of chemisorbed dyes.

**Table 3:** Calculated driving force of electron rejection and dye regeneration, $\Delta E_{\mathrm{CB}}$ and excited state lifetimes (τ).

Dyes	$E_{\mathrm{dye}}$ (eV)	$\lambda_{\max}$ (eV)	$E_{{\mathrm{dye}}^{*}}$ (eV)	$\Delta G_{\mathrm{inj}}$ (eV)	$\Delta G_{\mathrm{reg}}$ (eV)	$\mu_{\mathrm{normal}}$ (Debye)	$\Delta E_{\mathrm{CB}}$ (eV)	τ (ns)
ER	−5.30	2.57	−2.73	−1.27	−0.50	2.20	0.337	14.05
CA	−6.00	2.55	−3.45	−0.55	−1.20	7.77	0.410	15.20

**Table 4:** Calculated the static first hyperpolarizability of the two dyes in vacuum and solvent.

Condition	Dyes	$\beta_{xxx}$	$\beta_{xxy}$	$\beta_{xyy}$	$\beta_{yyy}$	$\beta_{xxz}$	$\beta_{xyz}$	$\beta_{yyz}$	$\beta_{xzz}$	$\beta_{yzz}$	$\beta_{zzz}$	$\beta_{\mathrm{tot}}$
vacuum	ER	4715.641	−146.531	−118.831	61.315	−28.349	−2.465	6.070	−102.532	−18.574	−20.764	4495.681
	CA	2199.959	−367.325	304.207	120.870	116.514	−8.887	−43.438	7.2242	−33.653	13.928	2528.461
solvent	ER	23111.926	−374.194	−533.766	127.514	149.044	297.519	47.8815	−202.700	54.757	−11.201	22377.028
	CA	2742.967	−1456.004	388.367	459.224	−303.563	−65.006	125.558	−90.987	−32.281	−99.709	3221.771

**Table 5:** Calculated transition properties of ethyl red (ER) and carminic acid (CA) adsorbed on TiO_2_ in ethanol.

Dyes	State	*E* (eV)	$\lambda_{\mathrm{abs}}$ (nm)	Contribution MO	Strength *f*
ER	S1	2.1153	586.13	(0.67992)H→L	0.3376
	S10	3.2715	378.98	(0.62913)H-2→L	0.2385
	S15	3.5159	352.64	(0.66223)H-1→L+4	0.0528
CA	S1	2.5252	490.98	(0.70029)H→L	0.2347
	S5	3.5909	345.28	(0.65835)H→L+1	0.2007
	S12	4.1885	296.01	(0.46691)H→L+3	0.3310

**Table 6:** Chemical reactivity of ethyl red (ER) and carminic acid (eV).

Dye	*A*	*I*	*h*	ω	ω^−^	*ω*^+^
ER	2.45	5.15	1.35	5.35	7.42	3.62
CA	3.36	5.78	1.21	8.63	11.07	6.50

## Supplementary Materials

The following are available online at www.mdpi.com/1996-1944/9/10/813/s1.
