# Supplementary material for: An Experimental and Theoretical Investigation of the Electronic Structures and Photoelectrical Properties of Ethyl Red and Carminic Acid for DSSC Application
Source: Materials (Basel). 2016 Oct 1;9(10):813. doi: 10.3390/ma9100813 (PMC5456617; doi:10.3390/ma9100813)
Supplement: Supplementary file 1 [file materials-09-00813-s001.docx]

Supplementary Materials: Experimental and theoretical investigation on the electronic structures and photoelectrical properties of Ethyl red and Carminic acid for DSSC application

Chaofan Sun, Yuanzuo Li, Peng Songand Fengcai Ma

Content:

**Table S1.** Experimental and calculated maximum absorption peaks (nm) of Ethyl red (ER) and Carminic acid (CA) with corresponding oscillator strengths by using different functional and basis set.

**Table S2.** Calculated HOMOs, LUMOs and energy gaps ($\Delta_{H-L}$) of two dyes in vacuum and solvent at the B3LYP/6-31G(d) level (eV).

**Table S3.** Calculated HOMOs, LUMOs and energy gaps ($\Delta_{H-L}$) of isolated dyes and dyes adsorbed on TiO_2_ in ethanol (eV).

**Table S4.** Calculated transition properties of Ethyl red (ER) adsorbed on TiO_2_ in ethanol.

**Table S5.** Calculated transition properties of Carminic acid (CA) adsorbed on TiO_2_ in ethanol.

**Figure S1.** FT-IR vibration analysis of Ethyl red (ER).

**Figure S2.** FT-IR vibration analysis of Carminic acid (CA).

**Figure S3.** Density of state and Partialdensity of state of Ethyl red (ER) adsorbed on TiO_2_.

**Figure S4.** Density of state and Partialdensity of state of Carminic acid (CA) adsorbed on TiO_2_.

**Table S1.** Experimental and calculated maximum absorption peaks (nm) of Ethyl red (ER) and Carminic acid (CA) with corresponding oscillator strengths by using different functional and basis set.

| **Table header** | **Table header** | **Cam-B3LYP** | **MPW1PW91** | **PBEPBE** | **LC-ωPBE** | **ωB97X** | **M062X** | **Exp.^a^** |
| --- | --- | --- | --- | --- | --- | --- | --- | --- |
| ER | 6-31G(d) | 375.29  (1.0763) | 407.53  (0.9207) | 469.23  (0.8011) | 349.20  (1.1746) | 356.26  (1.1482) | 376.98  (1.1616) | 502.50 |
|  | 6-31G(d,p) | 375.58  (1.0760) | 407.91  (0.9201) | 469.72  (0.8008) | 349.46  (1.1738) | 356.46  (1.1476) | 377.33  (1.1618) |  |
|  | 6-31+G(d,p) | 388.44  (0.8997) | 417.15  (0.6539) | 482.12  (0.6007) | 361.26  (1.1090) | 369.12  (1.0482) | 390.96  (1.1031) |  |
|  | 6-31++G(d,p) | 388.45  (0.9003) | 417.20  (0.6535) | 482.15  (0.6010) | 361.29  (1.1089) | 369.13  (1.0483) | 391.00  (1.1031) |  |
|  | 6-311++G(d,p) | 390.19  (0.9113) | 418.77  (0.6728) | 483.18  (0.6403) | 363.13  (1.1084) | 370.91  (1.0495) | 392.53  (1.0987) |  |
| CA | 6-31G(d) | 430.76  (0.3094) | 481.59  (0.2310) | 593.97  (0.1303) | 393.07  (0.3751) | 403.63  (0.3565) | 424.15  (0.3041) | 499.00 |
|  | 6-31G(d,p) | 430.72  (0.3109) | 481.50  (0.2323) | 593.34  (0.1311) | 393.10  (0.3769) | 403.70  (0.3583) | 424.35  (0.3055) |  |
|  | 6-31+G(d,p) | 432.89  (0.3220) | 483.48  (0.2396) | 594.56  (0.1235) | 394.87  (0.3913) | 405.90  (0.3722) | 426.73  (0.3144) |  |
|  | 6-31++G(d,p) | 432.95  (0.3224) | 483.52  (0.2398) | 594.56  (0.1233) | 394.93  (0.3914) | 405.96  (0.3725) | 426.79  (0.3147) |  |
|  | 6-311++G(d,p) | 432.95  (0.3224) | 485.78  (0.2331) | 597.00  (0.1245) | 397.04  (0.3830) | 407.99  (0.3642) | 427.73  (0.3086) |  |

^a^ maximum absorption peaks in experiment.

| 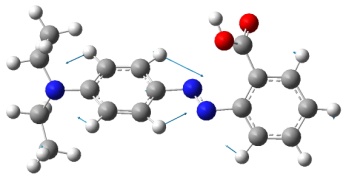  1148.33cm^−1^ | 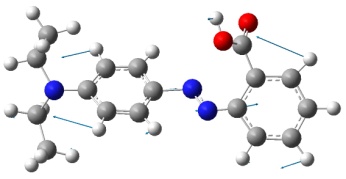  1267.70 cm^−1^ | 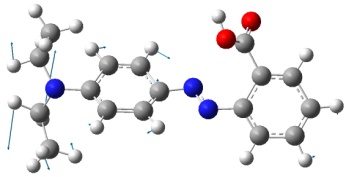  1354.54 cm^−1^ |
| --- | --- | --- |
| 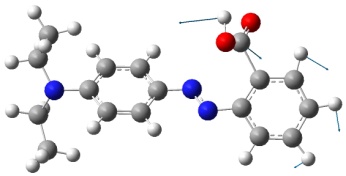  1383.86 cm^−1^ | 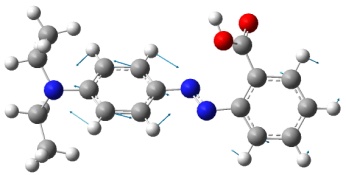  1600.81 cm^−1^ | 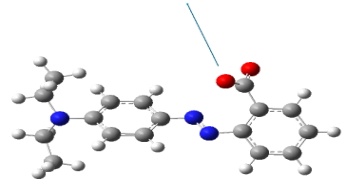  3435.10 cm^−1^ |

**Figure S1.** FT-IRvibration analysis of Ethyl red (ER).

| 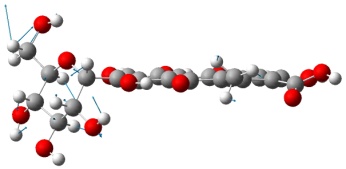  1081.97 cm^−1^ | 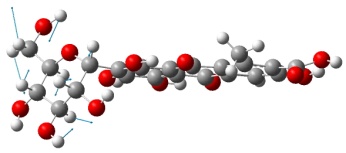  1249.99 cm^−1^ | 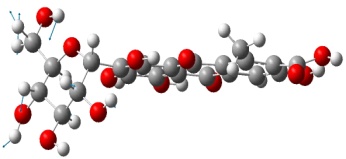  1446.34 cm^−1^ |
| --- | --- | --- |
| 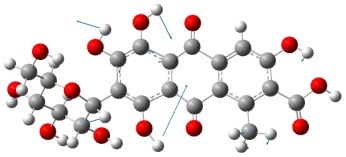  1574.08 cm^−1^ | 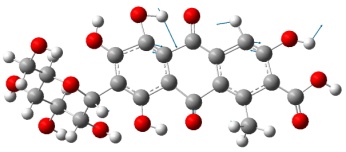  1621.35 cm^−1^ | 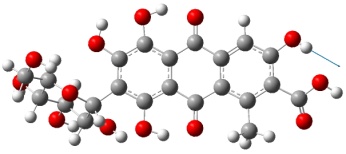  3439.51 cm^−1^ |

**Figure S2.** FT-IR vibration analysis of Carminic acid (CA).

**Table S2.** Calculated HOMOs, LUMOs and energy gaps ($\Delta_{H-L}$) of two dyes in vacuum and solvent at the B3LYP/6-31G(d) level (eV).

| **Dyes** | **HOMO** | **vacuum**  **LUMO** | $\boldsymbol{\Delta}_{\mathbf{H}\mathbf{-}\mathbf{L}}$ | **HOMO** | **solvent**  **LUMO** | $\boldsymbol{\Delta}_{\mathbf{H}\mathbf{-}\mathbf{L}}$ |
| --- | --- | --- | --- | --- | --- | --- |
| Ethyl red (ER) | −5.72 | −2.21 | 3.51 | −5.30 | −2.18 | 3.12 |
| Carminic acid (CA) | −6.25 | −3.31 | 2.94 | −6.00 | −3.12 | 2.82 |





**Figure S3.** Density of state and Partialdensity of state of Ethyl red (ER) adsorbed on TiO_2_.





**Figure S4.** Density of state and Partialdensity of state of Carminic acid (CA) adsorbed on TiO_2_.

**Table S3.**Calculated HOMOs, LUMOs and energy gaps ($\Delta_{H-L}$) of isolated dyes and dyes adsorbed on TiO_2_ in ethanol (eV).

| **Table header** | **HOMO** | **LUMO** | $\boldsymbol{\Delta}_{\mathbf{H}\mathbf{-}\mathbf{L}}$ |
| --- | --- | --- | --- |
| ER | −5.30 | −2.18 | 3.12 |
| ER/TiO_2_ | −5.71 | −2.47 | 3.24 |
| CA | −6.00 | −3.12 | 2.82 |
| CA/TiO_2_ | −6.01 | −3.15 | 2.86 |

**Table S4.** Calculated transition properties of Ethyl red (ER) adsorbed on TiO_2_ in ethanol.

| **State** | ***E* (eV)** | $\boldsymbol{\lambda}_{\mathbf{abs}}$ **(nm)** | **Contribution MO** | **Strength *f*** |
| --- | --- | --- | --- | --- |
| S1 | 2.1153 | 586.13 | (0.67992)H→L | 0.3376 |
| S2 | 2.3044 | 538.04 | (0.60535)H→L+1 | 0.0045 |
| S3 | 2.3078 | 537.25 | (0.59731)H-1→L | 0.0008 |
| S4 | 2.5166 | 492.68 | (0.66620)H→L+2 | 0.0357 |
| S5 | 2.5908 | 478.55 | (0.67106)H→L+3 | 0.0214 |
| S6 | 2.6068 | 475.62 | (0.69738)H-1→L+1 | 0.0022 |
| S7 | 2.8255 | 438.80 | (0.67523)H-1→L+2 | 0.0121 |
| S8 | 2.8895 | 429.08 | (0.68029)H-1→L+3 | 0.0023 |
| S9 | 3.1820 | 389.64 | (0.68113)H→L+4 | 0.0197 |
| S10 | 3.2715 | 378.98 | (0.62913)H-2→L | 0.2385 |
| S11 | 3.3716 | 367.74 | (0.64320)H-3→L | 0.0026 |
| S12 | 3.4311 | 361.36 | (0.67833)H→L+5 | 0.0094 |
| S13 | 3.4425 | 360.15 | (0.45190)H-2→L+1 | 0.0351 |
| S14 | 3.4594 | 358.39 | (0.53361)H-2→L+1 | 0.0524 |
| S15 | 3.5159 | 352.64 | (0.66223)H-1→L+4 | 0.0528 |
| S16 | 3.6715 | 337.69 | (0.50303)H-2→L+2 | 0.0435 |
| S17 | 3.6765 | 337.23 | (0.51138)H-5→L | 0.0121 |
| S18 | 3.7384 | 331.65 | (0.68747)H-1→L+5 | 0.0147 |
| S19 | 3.7538 | 330.29 | (0.59167)H-2→L+3 | 0.0390 |
| S20 | 3.7623 | 329.54 | (0.69132)H-3→L+1 | 0.0060 |
| S21 | 3.8157 | 324.94 | (0.63105)H→L+6 | 0.0313 |
| S22 | 3.8683 | 320.52 | (0.69385)H-4→L+1 | 0.0039 |
| S23 | 3.8954 | 318.28 | (0.45342)H→L+7 | 0.0076 |
| S24 | 3.9277 | 315.67 | (0.54986)H-7→L | 0.0003 |
| S25 | 3.9608 | 313.03 | (0.66954)H-3→L+2 | 0.0130 |
| S26 | 4.0057 | 309.52 | (0.67941)H-1→L+6 | 0.0089 |
| S27 | 4.0404 | 306.86 | (0.51154)H-8→L | 0.0044 |
| S28 | 4.0421 | 306.73 | (0.51414)H-3→L+3 | 0.0070 |
| S29 | 4.0758 | 304.20 | (0.49199)H-4→L+2 | 0.0406 |
| S30 | 4.1003 | 302.38 | (0.43136)H-4→L+2 | 0.0016 |

**Table S5.** Calculated transition properties of Carminic acid (CA) adsorbed on TiO_2_ in ethanol.

| **State** | ***E* (eV)** | $\boldsymbol{\lambda}_{\mathbf{abs}}$ **(nm)** | **Contribution MO** | **Strength *f*** |
| --- | --- | --- | --- | --- |
| S1 | 2.5252 | 490.98 | (0.70029)H→L | 0.2347 |
| S2 | 3.0969 | 400.35 | (0.67893)H-1→L | 0.0165 |
| S3 | 3.2528 | 381.16 | (0.52869)H-3→L | 0.0007 |
| S4 | 3.4622 | 358.11 | (0.64445)H-2→L | 0.1332 |
| S5 | 3.5909 | 345.28 | (0.65835)H→L+1 | 0.2007 |
| S6 | 3.6308 | 341.48 | (0.63418)H-9→L | 0.0003 |
| S7 | 3.8900 | 318.72 | (0.48573)H-4→L | 0.0398 |
| S8 | 3.9769 | 311.76 | (0.45035)H-6→L | 0.0718 |
| S9 | 4.0471 | 306.35 | (0.69867)H→L+2 | 0.0003 |
| S10 | 4.1380 | 299.62 | (0.37620)H→L+3 | 0.0581 |
| S11 | 4.1506 | 298.72 | (0.54628)H-5→L | 0.0160 |
| S12 | 4.1885 | 296.01 | (0.46691)H→L+3 | 0.3310 |
| S13 | 4.2434 | 292.18 | (0.54821)H→L+4 | 0.1805 |
| S14 | 4.2888 | 289.09 | (0.52236)H-7→L | 0.0056 |
| S15 | 4.3955 | 282.07 | (0.54902)H-8→L | 0.0022 |
| S16 | 4.4540 | 278.37 | (0.44918)H-3→L+1 | 0.0008 |
| S17 | 4.5053 | 275.19 | (0.52037)H→L+5 | 0.0257 |
| S18 | 4.5300 | 273.69 | (0.67422)H-1→L+2 | 0.0003 |
| S19 | 4.6163 | 268.58 | (0.66127)H-10→L | 0.0030 |
| S20 | 4.6372 | 267.37 | (0.45281)H-2→L+1 | 0.1363 |
| S21 | 4.6652 | 265.76 | (0.53280)H-1→L+3 | 0.0297 |
| S22 | 4.6803 | 264.91 | (0.36831)H-1→L+3 | 0.0317 |
| S23 | 4.7849 | 259.11 | (0.64120)H-1→L+4 | 0.0801 |
| S24 | 4.8486 | 255.71 | (0.60853)H-11→L+2 | 0.0059 |
| S25 | 4.9106 | 252.48 | (0.53925)H-6→L+1 | 0.1401 |
| S26 | 4.9438 | 250.79 | (0.65341)H→L+6 | 0.0038 |
| S27 | 5.0250 | 246.74 | (0.30610)H-12→L | 0.0012 |
| S28 | 5.0281 | 246.58 | (0.58037)H-2→L+2 | 0.0028 |
| S29 | 5.0491 | 245.56 | (0.38557)H-11→L+3 | 0.0013 |
| S30 | 5.0568 | 245.18 | (0.29614)H-13→L | 0.0071 |
